# Advances and challenges in Bioinformatics and Biomedical Engineering: IWBBIO 2020

**DOI:** 10.1186/s12859-023-05448-0

**Published:** 2023-10-18

**Authors:** Olga Valenzuela, Mario Cannataro, Irena Rusur, Jianxin Wang, Zhongming Zhao, Ignacio Rojas

**Affiliations:** 1 Faculty of Sciences, Applied Mathematics, University of Granada, Avenida de Fuente Nueva, 18071 Granada, Spain; 2 Data Analytics Research Center - Department of Medical and Surgical Sciences, University “Magna Græcia” of Catanzaro, Computer Engineering and Bioinformatics, Catanzaro, Italy; 3 Department of Informatic, University of Nantes, Rue de la Houssinière, BP 92208, 48565 Nantes, France; 4 Hunan Provincial Key Lab on Bioinformatics, School of Computer Science and Engineering, Central South University, Changsha, 410083 Hunan China; 5 Center for Precision Health, School of Biomedical Informatics and School of Public Health, University of Texas Health Science Center at Houston, Houston, TX 77030 USA; 6 Information Technology and Telecommunications Engineering, CITIC-UGR, University of Granada, Periodista Daniel Saucedo Aranda, 18071 Granada, Spain

**Keywords:** High performance in bioinformatics, Next generation sequencing and sequence analysis, Biomedicine, Biomedical engineering, Computational systems for modelling biological processes, Healthcare and diseases

## Abstract

This Supplement issue, presents five research articles which are distributed, mainly due to the subject they address, from the 8th International Work-Conference on Bioinformatics and Biomedical Engineering (IWBBIO 2020), which was held on line, during September, 30th–2nd October, 2020. These contributions have been chosen because of their quality and the importance of their findings. Those contributions were then invited to participate in this supplement for the following journals of BMC: BMC Bioinformatics and BMC Genomics. In the present Editorial in BMC journal, we summarize the contributions that provide a clear overview of the thematic areas covered by the IWBBIO conference, ranging from theoretical/review aspects to real-world applications of bioinformatic and biomedical engineering.

## Introduction to the IWBBIO 2020 edition

International Work-Conference on Bioinformatics and Biomedical Engineering (IWBBIO 2020) seeks to provide a discussion forum for scientists, engineers, educators and students about the latest ideas and realizations in the foundations, theory, models and applications in the field of bioinformatics and biomedical engineering.

It has been the first edition that has been held online, but due to the circumstances imposed by COVID-19, the safety and well-being of our participants were on top of the agenda. The conference was adapted to fulfill the regulations imposed by the competent authorities. The virtual presentations and the video conferences in real-time (using Zoom) were ultimately a great success and presented without major problems.

One of the main objectives of the conference is that research in the bioinformatic field can reach medical applications. The conference sought to focus on diverse fields to create multidisciplinary research integrating areas like biomedical engineering, computer since, mathematics, artificial intelligence, bioinformatics, statistics or biomedicine [1].

These ideas provided important advances to the scientific community in fields like genomics, next-generation sequencing, drug design and advanced pharmacology, biomedical modelling and e-health, among other.

The list of topics in the successive Call for Papers has also evolved, resulting in the following list for the present edition:

1. **Computational proteomics**. Analysis of protein-protein interactions, Protein structure modelling, Analysis of protein functionality, Quantitative proteomics and post translational modifications (PTMs), Clinical proteomics, Protein annotation, Data mining in proteomics.
2. **Next generation sequencing and sequence analysis**. De novo sequencing re-sequencing and assembly, Expression estimation, Alternative splicing discovery, Pathway Analysis, Chip-seq and RNA-Seq analysis, Metagenomics, SNPs prediction.
3. **High performance in bioinformatics**. Parallelization for biomedical analysis, Biomedical and biological databases, Data mining and biological text processing, Large scale biomedical data integration, Biological and medical ontologies, Novel architecture and technologies (GPU, P2P, Grid,etc.) for Bioinformatics.
4. **Biomedicine**. Biomedical Computing, Personalized medicine, Nanomedicine, Medical education, Collaborative medicine, Biomedical signal analysis, Biomedicine in industry and society, Electrotherapy and radiotherapy.
5. **Biomedical engineering**. Computer-assisted surgery, Therapeutic engineering, Interactive 3D modelling, Clinical engineering, Telemedicine, Biosensors and data acquisition, Intelligent instrumentation, Patient Monitoring, Biomedical robotics, Bio-nanotechnology, Genetic engineering.
6. **Computational systems for modelling biological processes**. Inference of biological networks, Machine learning in Bioinformatics, Classification for biomedical data, Microarray Data Analysis, Simulation and visualization of biological systems, Molecular evolution and phylogenetic modelling.
7. **Healthcare and diseases**. Computational support for clinical decisions, Image visualization and signal analysis, Disease control and diagnosis, Genome-phenome analysis, Biomarker identification, Drug design, Computational immunology.
8. **e-Health**. e-Health technology and devices, e-Health information processing, Telemedicine/e-Health application and services, Medical Image Processing, Video techniques for medical images, Integration of classical medicine and e-Health.

During IWBBIO 2020 several Special Sessions have been carried out. Special Sessions were a very useful tool in order to complement the regular program with new and emerging topics of particular interest for the participating community. Special Sessions that emphasize on multi-disciplinary and transversal aspects, as well as cutting-edge topics were especially encouraged and welcomed, and in this edition of IWBBIO 2020 were the following:

- **SS1.High-throughput Genomics: Bioinformatic Tools and Medical Applications. **
  Genomics is concerned with the sequencing and analysis of an organism’s genome. It is involved in the understanding of how every single gene can affect the entire genome. This goal is mainly afforded using the current, cost-effective, high throughput sequencing technologies.
  *Organizers: Prof. Dr. Cecilio Angulo, Prof. Dr. Juan Antonio Ortega,Prof. Dr. Luis Gonzalez*
- **SS2. Evolving Towards Digital Twins in Healthcare (EDITH).**
  Digital Twins is a very promising technique, as well as an ongoing research topic, imported from the Industry domain in order to develop Personalized Healthcare around the behavior of either patients’ disease or users’ health profile. The objective of this session is to present and discuss the advances in this important topic, Digital Twins, in the generation of knowledge. We advocates that this session will proportionate an important meeting point among different and variate researchers.
  *Organizers: Prof. M. Gonzalo Claros, Dr. Javier Pérez Florido, Dr. Francisco M. Ortuño*
- **SS3. Data Mining from UV/VIS/NIR Imaging and Spectrophotometry.**
  This special section provided discussion on novel development, implementation, and approaches in sensors, measurements, methods, evaluating software, and data mining focused on the spectral and color analysis. The topic should cover practical examples, strong results, and future visions.
  *Organizer: Dr. Jan Urban *
- **SS4: Intelligent Instrumentation.**
  Instruments and devices are almost similar and used for different scientific evaluations. They have become intelligent with the advancement in technology and by taking the help of artificial intelligence. In our daily life, sensors are corporate in several devices and applications for a better life. Such sensors as the tactile sensors are included in the touch screens and the computers’ touch pads. The input of these sensors is from the environment that converted into an electrical signal for further processing in the sensor system. The sensor’s main role is to measure a specific quantity and create a signal for interpretation.
  *Organizer: Prof. Dr. Barney *
- **SS5. Image Visualization and Signal Analysis.**
  Any signal that is transmitted from a biological or medical source can be referred to as a biosignal. On the other hand, medical imaging is the technique and the process of creating visual representations of the inside of the body for clinical analyzes and medical interventions as well as the visual representation of the function of some organs or tissues (physiology). The medical images also create a database of normal anatomy and physiology to identify anomalies. Although imaging of harvested organs and tissues can be done for medical reasons, such procedures are generally considered part of the pathology rather than medical images.
  *Organizers: Prof. Dr. L.Wang *
- **SS6. Analysis of Protein-protein Interactions. **
  Protein-protein interactions (PPI) are related to the association of proteins and the study of these associations from the perspective of biochemistry, signal transduction and protein interaction networks. Interactions between proteins are important in many biological processes.
  *Organizers: Dr. Yang *
- **SS7. Computational Approaches for Drug Design and Personalized Medicine.**
  With continuous advancements of biomedical instruments and the associated ability to collect diverse types of valuable biological data, numerous recent research studies have been focusing on how to best extract useful information from the ‘Big biomedical Data’ currently available.
  *Organizer: Prof. Dr. Hesham H. Ali*

## Contributions of this special issue

Those papers that were deemed particularly relevant, taking into account the evaluation and opinion of the reviewers and chairs, were then invited to participate in this supplement for the following BMC journals: BMC Bioinformatics and BMC Genomics.

The first paper authored by Dimitris Grigoriadis et al. [2], presented a novel Deep Learning-based method for effective removal of noisy CAGE signals. The distribution, abundance, and utilization of transcription start sites (TSS) within promoters is poorly understood. Cap Analysis of Gene Expression (CAGE) has become a popular protocol for gene expression profiling that quantifies the usage of TSS by detecting the 5’ end of capped RNA molecules. These results highlight the need for computational methods that can effectively remove the excessive amount of noise from CAGE samples, leading to accurate TSS annotation and quantification of promoter usage. Regardless of sample quality, there are a significant number of CAGE peaks that are not associated with transcription initiation events. Indeed, there are a growing number of studies in the literature suggesting that CAGE can also detect 5’-capping events that are byproducts of transcription.

This raises the need for computational methods that can accurately increase the signal-to-noise ratio in data from CAGE, leading to error-free annotation of transcription start sites (TSS) and quantification of regulatory region usage. In this paper, the authors presented DeepTSS, a novel computational method for processing CAGE samples that combines genomic signal processing (GSP), structural DNA features, evidence of evolutionary conservation, and raw DNA sequence with Deep Learning (DL) to provide predictions for a single nucleotide TSS with an unprecedented level of performance. DeepTSS outperformed existing algorithms on all benchmarks, achieving 98% precision and 96% sensitivity (accuracy 95.4%) on the protein-coding gene strategy, with 96.6% of positive predictions overlapping active chromatin and 98.3% and 92% colocalized with at least one transcription factor and H3K4me3 peak, respectively.

The article by Luca Cappelletti et al. [3] focused on the use of deep neural networks that can accurately predict active regulatory regions in specific cell lines. Noncoding DNA regions, which make up 98% of the total human genome, have historically been considered “junk DNA.” However, their importance is now recognised in the scientific community because noncoding cis-regulatory regions (CRRs) regulate the transcription of neighbouring genes and thus determine the spatio-temporal patterns of gene expression. The annotation and characterization of tissue-specific cis-regulatory elements (CREs) in non-coding DNA is an open challenge in computational genomics. Recent studies have shown that genetic variants occurring in CRRs are strongly correlated with pathogenicity or harmfulness.

Deep-learning techniques, have recently achieved cutting-edge results in this challenging computational task. In this study, the authors provided additional evidence that feed forward neural networks (FFNNs) can be trained on epigenetic data and one-dimensional convolutional neural networks (CNN) trained on DNA sequence data can successfully predict active regulatory regions in different cell lines. Authors showed that model selection using Bayesian optimization applied to both FFNN and CNN models can significantly improve the performance of deep neural networks by automatically finding models that best fit the data. Furthermore, they showed that techniques applied to balance active and inactive regulatory regions in the human genome in training and testing data can lead to overoptimistic or poor predictions. In this paper is recommended using actual unbalanced data that were not used to train the models to evaluate their generalization performance. The experimental results confirm that deep neural networks can accurately predict active regulatory regions in specific cell lines and that automatic model selection by Bayesian optimization improves the quality of the learner and that rebalancing of the data significantly affects the predictive performance of the models. Finally, the convolutional models achieve performance close to that of feed-forward models using epigenomic information.

Automatic annotation of protein functions is an important topic in the field of bioinformatics because protein annotation is inadequate due to the high cost and time-consuming manual procedures for function identification. To be useful, protein sequences must be annotated with functional properties such as Enzyme Commission (EC) numbers and Gene Ontology (GO) terms. The development of computational tools for automatic annotation that leverage the high-quality manual annotations already available in UniProtKB/SwissProt is an important research problem. In the paper of Bishnu Sarker et al. [4], the authors extend the GrAPFI (graph-based automatic protein function inference) method (Sarker et al. in BMC Bioinform 21, 2020; Sarker et al., in Proceedings of 7th international conference on complex networks and their applications, Cambridge, 2018), a Graph-based Automatic Protein Function Inference approach, to add to the GO annotation and rename it GrAPFI- GO.

The authors have presented a pruning technique based on semantic similarity to eliminate the outlier annotations and a hierarchical post-processing step to enrich the remaining annotations with term preprocessing. The authors proposed several types of similarity measures based on common neighbors in the graph. Moreover, the terms on GO are hierarchically arranged according to semantic parent–child relationships. Therefore, an efficient pruning and post-processing procedure that takes into account both semantic similarity and hierarchical relationships among GO terms has been presented. The authors produced experimental results comparing the GrAPFI-GO method with and without considering the similarity of common neighbors. They also tested the performance of GrAPFI- GO and other annotation tools for GO annotation on a benchmark of proteins with and without the proposed pruning and post-processing procedure. As conclusion, the authors highlight that the proposed semantic hierarchical post-processing can improve the performance of GrAPFI-GO and other annotation tools.

Bacterial typing is a technique used to distinguish between different strains within a species. Typing is an important tool in epidemiology as it helps to find sources of infection as they are transmitted, and it is also used for epidemiological surveillance. Typing methods such as pulsed field electrophoresis (PFGE) or multilocus sequence typing (MLST) are used in clinical practise. Unfortunately, the discriminatory power of these methods is not sufficient to distinguish closely related bacterial strains, and they should be combined with methods such as whole genome sequencing (WGS), which can even find single nucleotide variants. An alternative to these methods is mini-MLST, a rapid, inexpensive and robust method based on high-resolution enamel analysis. In the paper by Marketa Nykrynova et al. [5], the authors presented a pipeline for the detection of variable fragments in unmapped reads based on a modified hybrid assembly approach using data from a sequencing platform.

The authors demonstrated the ability to identify one variable fragment in de novo assembled scaffolds of 21 *Escherichia coli* genomes and three variable regions in scaffolds of 31 *Klebsiella pneumoniae* genomes. For each identified fragment, melting temperatures are calculated based on the nearest neighbor method to verify the discriminatory power of the mini-MLST. As the most important conclusion, the authors highlight that the identified variable regions can then be used in efficient laboratory methods for bacterial typing such as the mini-MLST with high discriminatory power and completely replace expensive methods such as the MLST. The results can and will be delivered in a shorter time, enabling immediate and rapid infection surveillance in clinical practice. A disadvantage of the proposed methods is the uncertainty in the data compiled de novo.

## Conclusions and Acknowledgement

The articles presented in this special issue provide some recent progresses in to Bioinformatics and Biomedicine Engineering fields. As Guest editors, we would like to express our thankfulness to all the authors who contributed their high quality research to the achievement of this supplement. Also, we are very grateful to expert scientists that have actively collaborated with their recommendations and suggestions to review and improve these contributions. We specially thank to Mr. Omar El Bakry for his excellent and constant support with the publication and edition of this supplement. It has been an honor for us to participate in it.

We finally invite authors and readers of this supplement to submit their recent works to future editions of IWBBIO, which will be announced at https://iwbbio.ugr.es. We wish the readers can benefit from insights of these relevant papers, and contribute to these rapidly and dynamics growing areas.

## Data Availability

Not applicable.
